# Case of Complete Division of the Tendo Achillis, by the Blow of an Axe

**Published:** 1832

**Authors:** John L. Richmond

**Affiliations:** Cincinnati


ART. IV. *Case of Complete Division of the Tendo Achillis by the blow of an Axe.* Communicated by JOHN L. RICHMOND, M. D. of Cincinnati.

On the 22d of September, 1829, Isaac Allen, about 19 years of age, received the full blow of a heavy sharp chopping axe directly across the tendo Achilles: the force of the blow was such as to divide every thing posterior to the bones, and marking the tibia considerably, to pass obliquely downwards about one-fourth through the malleolus of the Fibula.

He was taken up, carried to the house, and laid on a straw bed, *very prudently*, with his foot considerably elevated, and wrapped up in several thicknesses of cloth. On unwrapping the ankle, the blood flowed, *pulsatim* from two arteries.

My first object, of course, was to secure the arteries; which was attended with no difficulty, and requires no detail. The next object was to dress the wound in such a manner as to secure adhesion by the first intention, and to secure, if possible, an adhesion of the tendon. For this purpose two stitches were applied to assist the adhesive straps in supporting the external parts. A patch of adhesive plaster was then spread of sufficient size and of a proper shape to cover the whole belly of the gastrocnemii muscles, leaving the lower end of my cloth for about two inches uncovered with plaster, then applying a slipper to the foot, with a very stiff sole, extending about an inch back of the heel, I flexed the knee, and extended the foot as much as was practicable without constraint—then passing a lacing string four times back and forward from the hind end of the slipper to the lower end of the plaster cloth, I drew them as nearly together as was necessary to bring the two ends of the divided tendon in perfect contact. The string was then fastened, and to conclude the whole, an elastic corset was bound to the instep, to prevent any flexion of the foot. In this situation it remained until the proper time for removing the sutures, at which time, no part of the dressings were removed except what was necessary to enable me to remove the sutures. The dressings were then reapplied and caused to remain until time for the ligatures to come off from the arteries, when they were removed, and the dressing reapplied and continued about four days longer. They were then carefully removed, the foot rubbed, but not suffered to be flexed, nor the knee extended; dressings were again applied as before, and continued uninterrupted for two weeks longer. The incision adhered, perfectly, by the first intention, and to my great satisfaction, on removing the dressing, I found that the tendon had *perfectly* adhered. Owing, however, to the long confinement, which had been between three and four weeks, he was unable, at first, to flex the foot or extend the knee. This difficulty, however, subsided by degrees, the adhesion of the tendon remained perfect, and in a few weeks he acquired as perfect a use of the foot as he ever had, and now at the end of three years, he experiences no kind of inconvenience from having received the cut. He is a great fellow for hopping, and as that was the foot which he formerly used for that purpose, he continues to use it, and that without the least impediment. This is the most perfect cure which I have ever witnessed of entire division of that tendon.

What I consider of the greatest importance in this case, and the principal reason why I report it, is the application of the adhesive plaster to the calf of the leg, by which means the muscles were so perfectly drawn down as to produce the most natural coaptation of the ends of the divided tendon, and that without obstructing the venous circulation. At the same by the firm and equable support of the belly of the muscle, the tendency to twitching and spasmodic contraction, which is a very great obstacle to the preservation of the divided extremities of the tendon in juxta position, was effectually obviated.

I have witnessed the application of the dressing connected with a garter or fillet, *around the leg;* but I have never seen it succeed, without producing an enlargement of the tendon at the point of separation, nor have I ever seen another case in which there was not some impediment in the motions of the foot and a weakness of the ancle; but in this case there is neither. In the application of the fillet, there is also some inconvenience arising from the obstruction of the venous circulation, which, by the application of the adhesive plaster is entirely avoided. It is necessary, however, to observe, that it should be so applied as to include the whole belly of the gastrochnemii muscles, and to compress them smartly, in order to bring down the muscles sufficiently to bring the ends of the separated tendon in perfect contact.

It should, moreover, never be forgotten, that the other part of the incision having adhered by the first intention, is no proof that the tendon has adhered also; this would be impossible. If a tendon adheres in three weeks by the first intention, its progress is comparatively as favorable, as a union of cellular substance and muscle in three or four days; no trial, therefore, should be made short of three weeks, for fear of losing what is already gained.

*September* 4, 1832.

